# Establishing conditions for the generation and maintenance of estrogen receptor-positive organoid models of breast cancer

**DOI:** 10.21203/rs.3.rs-3341539/v1

**Published:** 2023-10-04

**Authors:** Michael UJ Oliphant, Dipikaa Akshinthala, Senthil K Muthuswamy

**Affiliations:** Harvard Medical School; National Cancer Institute, National Institute of Health; National Cancer Institute, National Institute of Health

## Abstract

Patient-derived organoid models of estrogen receptor-positive (ER+) breast cancer would provide a much-needed tool to understand drug resistance and disease progression better. However, the establishment and long-term maintenance of ER expression, function, and response in vitro remains a significant challenge. Here, we report the development of an ER+ breast tumor organoid medium (BTOM-ER) that conserves ER expression, estrogen responsiveness, and dependence, as well as sensitivity to endocrine therapy of ER+ patient-derived xenograft organoids (PDXO). Our findings demonstrate the utility of subtype-specific culture conditions that better mimic the characteristics of the breast epithelial biology and microenvironment, providing a powerful platform for investigating therapy response and disease progression of ER+ breast cancer.

## Introduction

Breast cancer remains one of the most frequently diagnosed cancers and causes of cancer deaths among women worldwide(Siegel et al., 2022) Despite estrogen receptor-positive (ER+) breast cancer being the most prevalent subtype, the establishment of clinically relevant models remains a challenge. More recently, three-dimensional (3D) organoid models established from patient tumors bridge the gap between cell culture and patient-derived xenograft platforms (Dekkers et al., 2021; Guillen et al., 2022; Sachs et al., 2018). Previous studies have shown that organoid media formulations are critical for the establishment, growth, and maintenance of patient-specific characteristics of ER + breast cancer organoid models(Guillen et al., 2022; Hogstrom et al., 2023; Sachs et al., 2018). However, establishment, decreased estrogen receptor (ER) expression, and loss of ER-dependent transcription after extended culturing continue to be a challenge for the generation and use of organoid models from ER + breast tumors.

Here, we report the development of a simplified media using breast tissue-relevant cytokines and growth factors to establish and expand ER + organoids that retain ER expression over long-term culture. In addition, they retain responsiveness to estrogen and sensitivity to the anti-hormone therapeutic agent, fulvestrant, identifying a new approach for generating ER + organoid models for breast cancer.

## Results

### Optimization of media conditions that support robust growth of ER+ PDX Organoids

In designing the media, we excluded serum to prevent the proliferation of fibroblasts and stem cell factors to prevent reprogramming or the expansion of undifferentiated cells at the expense of differentiated epithelial cells. In particular, R-spondin-1 was not included due to its roles in promoting stem and basal epithelial cell differentiation and proliferation (Broutier et al., 2016; Jardé et al., 2016), and Noggin for its role in promoting stemness by interfering with ER transcriptional responses (Páez-Pereda et al., 2003). Bovine pituitary extract (BPE) and B27 were used as a serum-free mitogenic supplement (Hammond et al., 1984; Maciag et al., 1981) and to protect against oxidative stress (Kent & Bomser, 2003). We included growth factors implicated in ER biology in developmental biology studies. For example, prolactin stimulates ER expression and works in concert with progesterone receptor to promote autocrine secretion-mediated mammary epithelial cell and breast cancer proliferation (Frasor & Gibori, 2003; Gutzman et al., 2004; Naylor et al., 2003). Amphiregulin supports ER expression and estrogen signaling in the mammary gland (Ciarloni et al., 2007). IL6 expression is positively correlated with hormone receptor-positive tumors and has been shown to coordinate estrogen expression in vivo (Fontanini et al., 1999; Purohit et al., 2002; Sasser et al., 2007; Speirs et al., 2000). The above factors were added to DMEM/F12 media in conjunction with other growth factors frequently used to support the proliferation of mammary epithelial cells in culture and *in vivo*, including FGF2, FGF10, EGF, insulin, and hydrocortisone (See Suppl.1 for details of media recipe and preparation). Supplementation of estradiol to the media caused varying effects on cell growth; some organoid cultures showed no difference, whereas others showed a non-significant increase in growth (Suppl. Fig. 1). Furthermore, estrogen receptor expression and activity can be negatively regulated by prolonged exposure to E2 in culture and *in vivo* (Bondar et al., 2009; Borrás et al., 1994; Hatsumi & Yamamuro, 2006); hence no E2 supplementation was used for routine culture.

**Figure 1:**
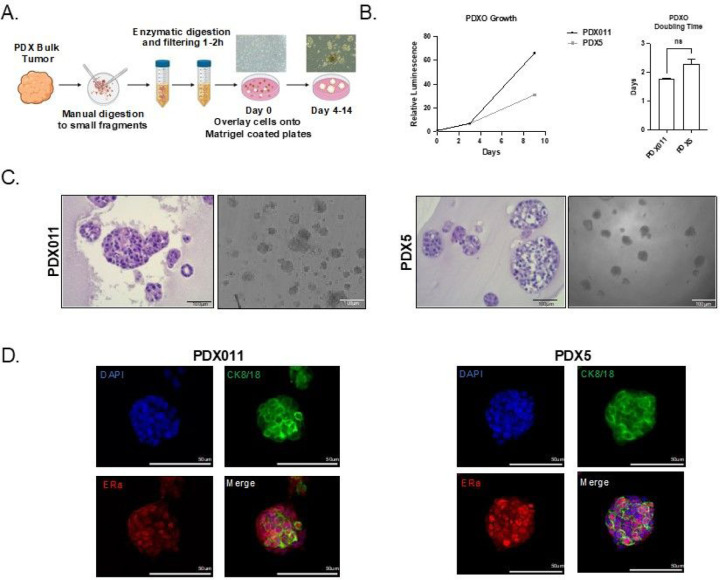
BTOM promotes robust growth and ER expression of ER+ PDXOs. A) Schematic describing establishment of PDXOs. B) A representative experiment of growth over time of PDX011 and PDX5 organoids, as measured by 3D Cell Titer Glo (Promega). Doubling time was calculated using GraphPad Prism Software. C) H&E and brightfield images to show the morphology of established PDXOs. Scale bars: 100um D) Immunofluorescence of PDXOs displaying CK8/18 and ER-alpha expression. DAPI was used to stain nuclei.

We used ER+ patient-derived xenograft tumors (Guillen et al., 2022) (Table 1) to find the optimal concentrations of growth factors and defined a Breast Tumor Organoid Media for ER+ breast cancers (BTOM-ER) (Fig 1a, Table 2). The PDXOs achieved robust growth with an average doubling time of 2.042 days (Fig 1b) and remained stable in later passages. The phase and histomorphology of PDXOs were heterogeneous within cultures consisting of solid and hollow organoids with the formation of budding structures between 10–14 days post-plating (Fig 1c). Both proliferation rates and phase morphological characteristics were relatively consistent over 15 passages, as determined by phase morphology and Incucyte live-cell imaging (data not shown). Importantly, immunofluorescence analysis for cytokeratin 8/18 and estrogen receptor demonstrated the presence of ER+ luminal tumor epithelia (Fig 1d). Collectively, these data suggest that BTOM-ER allows for the establishment of ER+ luminal epithelial organoid cultures that maintain proliferative capacity during long-term culture.

### PDXOs maintain estrogen receptor expression, are estrogen-responsive, and are sensitive to endocrine therapy.

**Figure 2:**
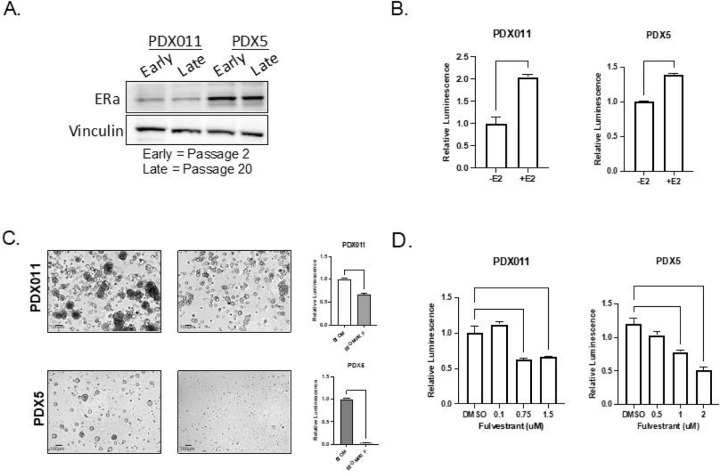
PDXOs grown in BTOM maintain estrogen-dependent phenotypes of ER+ breast cancer. A) Western blot showing Estrogen Receptor alpha expression in PDX011 and PDX5 organoids. VINCULIN was used as a loading control. B) Luminescence levels in PDX011 and PDX5 organoids after 10 days of treatment with b-estradiol or vehicle control (EtOH). N=3. C) Representative brightfield images of Day 10 PDX011 and PDX5 grown in either BTOM or phenol red-free BTOM (left). Luminescence values for PDX011 and PDX5 organoids 10 days after being grown in either BTOM or phenol red-free BTOM (N=3). D) Luminescence values for PDX011 and PDX5 after being treated for 5 days with increasing doses of fulvestrant or vehicle control (N=3).

Consistent with ER expression detected in the PDX tumors *in vivo*, ERalpha was detectable in both early (passage 2) and late passage (passage 20) organoid cultures (Fig 2a). Additionally, treatment of PDXOs with physiological estrogen levels resulted in increased proliferation compared to vehicle control (Fig 2b). In contrast, culturing the organoids in phenol-red-free media to exclude the weak estrogenic activity of phenol-red reduced cell proliferation in PDX5 tumor-derived cultures (Guillen et al., 2022) (Fig 2c), consistent with previous studies showing that these PDXs are responsive to estrogen stimulation (Gou et al., 2021; Guillen et al., 2022). Organoid lines from PDX011 tumors expressing wild-type ER showed only a weaker but significant decrease in proliferation. Finally, we treated PDXOs with the selective estrogen receptor degrader (SERD) fulvestrant, which is a widely used treatment for ER+ breast cancer. As expected, fulvestrant treatment resulted in a significant decrease in proliferation compared to vehicle control, suggesting that PDXOs require ER function to proliferate (Fig 2d). Thus, we demonstrate that ER+ PDXOs maintain ER expression, estrogen dependence, and responsiveness and retain endocrine sensitivity during long-term culture.

## Discussion

We report the development of an organoid culture media for the generation and maintenance of ER+ breast tumor organoids. We included growth factors and cytokines such as prolactin, amphiregulin, IL6, and bovine pituitary extract with established roles in ER biology and maintenance of ER expression. We excluded regulators of stemness, including WNT, R-Spondin-1, and Noggin, to avoid the expansion of stem-like epithelial lineages at the expense of differentiated lineages. Given the consideration of growth factors that impact ER biology, it is likely BTOM-ER will be efficient in supporting the establishment and maintenance of ER+ luminal epithelial organoids from normal breast or mouse mammary glands; however, further studies will be required to investigate this possibility. Similar to many other tumor organoid platforms, the organoid culture conditions outlined here do not contain fibroblasts or immune cells, which have been shown to play a significant role in ER-mediated transcription and endocrine therapy response (Brechbuhl et al., 2017; Place et al., 2011). However, the culture conditions reported here are suitable for co-cultures, as we demonstrated recently for the culture of T cells with mouse or rat mammary tumor organoid models to investigate T-cell mediated killing of tumor epithelia (Gil Del Alcazar et al., 2022; Meng et al., 2021 ). I should also be suitable to study the interaction between ER+ tumor-epithelia and stroma (Hogstrom et al., 2023). Thus, we believe that the culture conditions reported here can serve as a new platform for understanding ER biology in primary breast tumor-derived epithelia and for the development of ways to overcome drug resistance in ER+ breast cancer.

## Methods (please see Supplementary Materials 2 for further details)

### ER+ Breast Tumor Organoid Media (BTOM-ER)

For BTOM-ER growth media, 2.145 ml of Reagent A, 50 ml of Reagent B, and 1.0% penicillin-streptomycin were added to 100 ml of DMEM/F-12. Please see Table 1 for components of Reagent A and Reagent B, and Supplemental Material 1 for further details on components and media recipes.

### Estrogen Responsiveness and Dependence of PDXOs

For both estrogen responsiveness and dependence, organoids were digested and plated as above 40–50,000 cells/ml, depending on the organoid line. Three days after plating, media was refreshed, and wells were treated with either 1.0 nM of b-estradiol or vehicle control (EtOH) using the Tecan D300e drug dispenser. For dependence, media was replaced with fresh BTOM-ER or phenol red-free BTOM-ER. Media was refreshed every 2–3 days. After 10 days, organoids were assessed for viability using 3D Cell Titer Glo (Promega). Viability measurements were analyzed using GraphPad Prism software.

### Endocrine Therapy Treatment of PDXOs

Organoids were prepared as above, and on day three, media was refreshed, and wells were treated with either vehicle control or indicated concentrations of fulvestrant (Selleckchem) using the Tecan D300e drug dispenser. Media was refreshed, and plates were retreated every 2–3 days. After 5 days of treatment, organoids were assessed for viability using 3D Cell Titer Glo (Promega).

### Statistics and reproducibility

Error bars were generated by Standard Error Mean (SEM) calculations. For experiments with two conditions, an unpaired one-tailed Student’s T-test was performed. For experiments with three or more conditions, one-way ANOVA followed by a Bonferroni comparison was used. Doubling time was calculated by applying a non-linear regression for exponential growth for each condition.

## Supplementary Material

Supplement 1

## Figures and Tables

**Table 1.** PDXO Clinical Information

Patient ID	ER	PR	HER2	ER Mutation Status	Additional genetic alterations	Morphology
**PDX011**	+	+	−	WT	N/A	Solid spheres, with branching for larger organoids
**PDX5**	+	+	+	L536P	RBM10 and RNF111 deletions	Solid spheres with small numbers of protruding cells

**Table 2:** Components of BTOM-ER growth media

	Supplements/Growth factors	Vendor	Catalog #	Final concentration
**Reagent A**				
1	Bovine Pituitary Extract	Hammond cell tech	1078-NZ	0.8 ml for 100 ml
2	B27	Thermo	17504001	1.0 ml for 100 ml
3	Recombinant Human FGF- Basic (FGF2)	Peprotech	AF-100- 18B	10 ng/ml
4	Recombinant Human FGF10	Peprotech	100-26	10 ng/ml
5	Recombinant Human EGF	Peprotech	AF-100-15	2.0 ng/ml
6	Recombinant Human IL6	Peprotech	200-06	100 ng/ml
7	Recombinant Human Amphiregulin	Peprotech	100-55B	100 ng/ml
8	Recombinant Human Prolactin	Peprotech	100-07	10 ng/ml
9	Human Insulin	Sigma	I2643- 250MG	10 mg/ml
**Reagent B**				
	Hydrocortisone	Sigma	1316004-200Mg	0.5 mg/ml
