# Supplementary material for: Establishing conditions for the generation and maintenance of estrogen receptor-positive organoid models of breast cancer
Source: Res Sq. 2023 Oct 4:rs.3.rs-3341539. Preprint. [Version 1] doi: 10.21203/rs.3.rs-3341539/v1 (PMC10602055; doi:10.21203/rs.3.rs-3341539/v1)
Supplement: Supplement 1 [file NIHPPrs3341539v1-supplement-1.pdf]

Supplementary Files

This is a list of supplementary files associated with this preprint. Click to download.

- Suppl.materials.docx
